# Identifying Robust Radiomics Features for Lung Cancer by Using In-Vivo and Phantom Lung Lesions

**DOI:** 10.3390/tomography7010005

**Published:** 2021-02-09

**Authors:** Lin Lu, Shawn H. Sun, Aaron Afran, Hao Yang, Zheng Feng Lu, James So, Lawrence H. Schwartz, Binsheng Zhao

**Affiliations:** Department of Radiology, New York Presbyterian Hospital, Columbia University Medical Center, New York, NY 10032, USA; ll2860@cumc.columbia.edu (L.L.); shs2179@cumc.columbia.edu (S.H.S.); aafran@bu.edu (A.A.); yh2588@cumc.columbia.edu (H.Y.); zlu@radiology.bsd.uchicago.edu (Z.F.L.); js998@cumc.columbia.edu (J.S.); lhs2120@cumc.columbia.edu (L.H.S.)

**Keywords:** radiomics, reproducibility, robustness, NSCLC, phantom, EGFR

## Abstract

We propose a novel framework for determining radiomics feature robustness by considering the effects of both biological and noise signals. This framework is preliminarily tested in a study predicting the epidermal growth factor receptor (EGFR) mutation status in non-small cell lung cancer (NSCLC) patients. Pairs of CT images (baseline, 3-week post therapy) of 46 NSCLC patients with known EGFR mutation status were collected and a FDA-customized anthropomorphic thoracic phantom was scanned on two vendors’ scanners at four different tube currents. Delta radiomics features were extracted from the NSCLC patient CTs and reproducible, non-redundant, and informative features were identified. The feature value differences between EGFR mutant and EGFR wildtype patients were quantitatively measured as the biological signal. Similarly, radiomics features were extracted from the phantom CTs. A pairwise comparison between settings resulted in a feature value difference that was quantitatively measured as the noise signal. Biological signals were compared to noise signals at each setting to determine if the distributions were significantly different by two-sample *t*-test, and thus robust. Four optimal features were selected to predict EGFR mutation status, Tumor-Mass, Sigmoid-Offset-Mean, Gabor-Energy and DWT-Energy, which quantified tumor mass, tumor-parenchyma density transition at boundary, line-like pattern inside tumor and intratumoral heterogeneity, respectively. The first three variables showed robustness across the majority of studied CT acquisition parameters. The textual feature DWT-Energy was less robust. The proposed framework was able to determine robustness of radiomics features at specific settings by comparing biological signal to noise signal. Identification of robust radiomics features may improve the generalizability of radiomics models in future studies.

## 1. Introduction

Since its conception, radiomics [1,2] has made an enormous impact on establishing medical images as quantitative data and led to the creation of algorithmic models capable of performing diagnostic [3,4], prognostic [5,6], histological [7,8,9], and even genomic classification [10,11]. However, reproducibility and repeatability of radiomics features and generalizability of radiomics models have been sources of concern and a major barrier to clinical translation [12,13]. In addition to reproducibility and repeatability, identifying robustness is crucial to finding features and building models that will withstand the test of external validation [14].

CT scan parameters are known to affect radiomics features, but the details are complex [12,15,16]. For example, we have shown that in general, radiomics features can be more reproducible between images of different slice thicknesses than at different reconstruction kernels [12,15]. This also changes depending on the type of radiomics feature and the specific scanning parameter called into question. In addition to slice thickness and reconstruction kernel, tube current, radiation dose, and many other parameters have also been studied for their impact on radiomics feature reproducibility [16,17,18].

It has been suggested that only reproducible features be included in training predictive models [19]. Current methods of establishing the reproducibility of radiomics features revolve around using test-retest analysis on phantoms or patients [15,18]. However, it is difficult to capture all of the variety and variability of CT scanning and acquisition parameters and their effects on radiomics features in a dedicated reproducibility study. In addition, there is disagreement between studies on the reproducibility of features and whether slice thickness or reconstruction kernel has a stronger effect on these features [17]. Given the variety of acquisition settings and the ethical implications of obtaining multiple repeat CT scans on patients, phantom models may be preferable to human subjects for radiomics studies for current CT scanners and for future generations of scanners.

Previous reproducibility and robustness studies have focused on the effect of different scanning settings on radiomics features, also known as the random noise generated from CT acquisition parameters. However, this only describes the consistency of radiomics features relative to itself, that is, if the random noise is kept under a certain threshold. This adequately demonstrates reproducibility, but does not provide any information on whether a feature can still distinguish two classes under different circumstances. For example, a previous radiomics study on liver nodule classification from abdominal CT scans showed that a sizable number of features could be significantly influenced by the quality of image contrast-enhancement [20], i.e., the noise introduced by variations in contrast-enhancement may more significantly alter the overall imaging phenotype than the underlying change of tumor biology. In order to determine robustness, there is a need to consider the magnitude of noise relative to the biological signal and exclude features that are more influenced by noise than biology.

Therefore, we propose a new framework for determining the robustness of radiomics features by assessing the relationship between biological and noise signals. If the characterized signal between two biological groups is significantly higher than the noise signals generated across different CT acquisition parameters, the radiomics feature was determined to be robust. Furthermore, we investigated the usefulness of the proposed framework in a real medical application by evaluating reproducible radiomics features to non-invasively predict epidermal growth factor receptor (EGFR) mutant status in non-small cell lung cancer (NSCLC) patients.

## 2. Methods

### 2.1. Study Design

**Figure 1:**
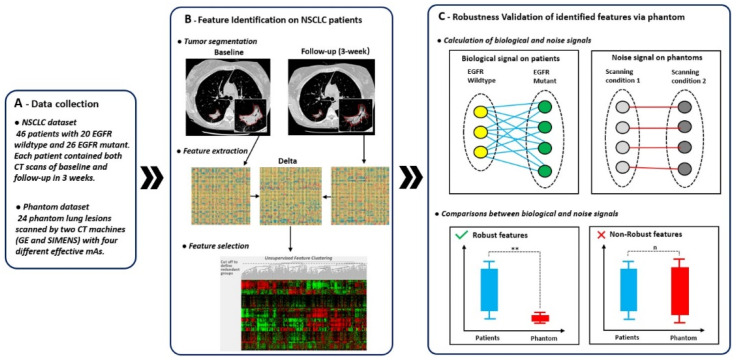
Overview of study design. The overall study design consisted of three parts: (**A**) data collection, (**B**) feature identification on non-small cell lung cancer (NSCLC) patients, and (**C**) robustness validation of identified features via phantoms. In (**C**), lines between elements represented absolute difference of feature value between two elements.

The overview of our study design is presented in Figure 1. Our work consisted of three parts: (A) data collection, (B) feature identification on NSCLC patients, and (C) robustness validation of identified features via phantoms. Our main innovation was the third part which involved the calculation and comparison of biological and noise signals. We defined the biological signal as the difference between patient groups of EGFR wildtype and mutant and the noise signal as the difference of phantom lesions between two different scanning conditions.

### 2.2. Patient and Phantom Data

The imaging data of 46 patients were collected in a previous clinical trial (NCT00588445), “Prospective trial with preoperative gefitinib to correlate lung cancer response with EGFR exon 19 and 21 mutations and to select patients for adjuvant therapy” that was IRB approved [21]. In this study, only fully de-identified data were used for the analysis. As only de-identified imaging data were made available for this study, it is not considered a human study. Detailed characteristics of patients can be found in previously published literature [22], which utilized this patient data to investigate the value of early change in tumor volume as an imaging biomarker for response assessment.

Patients were included if they had a smoking history of less than 15 pack years, or if their tumors had histologic features suggestive of bronchioalveolar cancer. They received gefitinib daily for 3 weeks before surgery and discontinued it 2 days before their operation. The EGFR mutation status of resected tumor tissues were analyzed and determined using a PCR-based method described in a previous publication [21]. Among the 46 patients, 20 patients had tumors with EGFR wildtype and 26 patients had tumors with EGFR mutants (primary tumor, one per patient). Baseline scanning was done within 2 weeks before gefitinib initiation and a single follow-up scanning was done prior to surgery and about three weeks post therapy. Baseline and follow-up non-contrast enhanced CT imaging were performed for all patients using a LightSpeed 16 scanner (GE Medical Systems, Milwaukee, WI) during a breath-hold. Both baseline and follow-up scanning were reconstructed into CT images with 1.25 mm slice thickness and a sharp reconstruction kernel.

The phantom images used in this study came from an anthropomorphic thoracic phantom that had been customized by the Quantitative Imaging Biomarkers Alliance (QIBA) task group led by FDA scientists [23] and acquired at Columbia University Vagelos College of Physicians and Surgeons [24]. A subset of lung phantom lesions which consisted of 24 lesions with two different sizes (10 and 20 mm effective diameter), four different shapes (spherical, elliptical, lobular, and spiculated) and three different densities [−630, −10, and +100 Hounsfield Unit (HU)] were used. A detailed description of the used phantom lesions can be found in a previous preliminary study [24], within which the phantom was used to explore variability in CT characterization of tumors. The scanning protocols of the phantom are listed in Table 1. Two scanners, Scanner #1 (GE Light Speed Pro) and Scanner #2 (SIEMENS Sensation 16), were used to scan the phantom with varying effective mAs and fixed reconstruction kernel, slice interval and thickness, pixel spacing and tube voltage.

The scanning conditions used for the phantom settings were varied by using an x-ray tube current of 395, 195, 100, and 50 mA for Scanner #1 and 480, 260, 130, and 65 mA for Scanner #2. The exposure time was 0.7 s for Scanner #1 and 0.5 s for the Scanner #2. These settings were chosen to obtain roughly equivalent tube current-time product (mAs), which is proportional to the radiation output, between the two scanners in order to make fair comparisons [25]. As a result, the conditions varied can be narrowed down to 4 roughly equivalent tube current-time products, or effective mAs, between the 2 different scanners.

### 2.3. Radiomics Feature Identification on NSCLS Patients

The identification of radiomics features mainly consisted of three procedures: (1) tumor segmentation, (2) feature extraction and (3) feature selection.

First, an experienced radiologist with more 25 years of experience in chest CT interpretation segmented each lesion on both baseline and follow-up CT images by using an imaging platform that integrated a semi-automated lung nodule segmentation algorithm [26,27]. The radiologist was blinded to mutation status. Each nodule was segmented on the CT images under the lung (width, 1500 HU; level, −500 HU) and mediastinal (width, 400 HU; level, 80 HU) window/level settings. Phantom and patient lung lesions were segmented using the same procedure.

Second, an in-house feature extraction software, implemented on the Matlab platform (version 2017b, Mathworks, Natick, MA, USA), was used to extract the 1160 quantitative features from both baseline and follow-up from each segmented tumor [12]. In particular, to characterize tumor response phenotypes, delta features were used. The delta feature values were defined as the baseline feature values minus the follow-up feature values [28] (See Figure 1, part B). These delta features characterized tumor phenotypic changes in terms of size (e.g., volume, largest diameter), boundary sharpness (e.g., sigmoid slope), shape (e.g., eccentricity, compactness), and texture patterns (e.g., gray level co-occurrence matrix (GLCM)). The implementation of these features is detailed in previous publications [7,8,12].

Third, the selection of radiomics features consisted of two steps: (1) remove non-reproducible features, (2) determine non-redundant and informative features. First, reproducibility analysis on test-retest CT dataset [29] were performed, and features with a concordance correlation coefficient smaller than 0.85 were excluded [12]. Second, a hierarchical cluster analysis was performed to determine non-redundant and informative features [15,18,30]. The hierarchical cluster analysis consisted of three steps: (1) calculate spearman’s rank correlations between features, (2) organize features into clusters using unsupervised hierarchical clustering based on feature correlations, i.e., features with correlation larger than 0.85 were assigned to the same clustering group, and (3) univariance analysis based on area under the receiver operating curve (AUC) were performed. Only features with the highest AUC within each cluster that also had an AUC larger than 0.7 were selected as the representatives, i.e., the non-redundant and informative features.

### 2.4. Robustness Validation of Robust Features via Phantoms

We defined robustness as a feature’s ability to be free from the influence of random noise. In this case, random noise is the variability in feature value due to different imaging conditions. In order to not be impacted by the noise, a feature’s biological signal must significantly outweigh random variability. The robustness validation consisted of two steps, calculation of biological and noise signals and comparison between the two.

First, the biological signals (BS) were calculated by measuring the differences in feature values between the two groups, which in this case are lung lesions that are EGFR mutants versus lung lesions that are EGFR wildtype, as defined in Equation (1) (See Figure 1, part C). The noise signals (NS) were calculated by measuring the difference in feature values solely from using a different scanner or tube current, as defined in Equation (2) (See Figure 1, part C).

(1) $$BS=\{d|d=dist\left(x_{ri},x_{sj}\right),i\in \left(1,\ldots ,n_{r}\right),j\in \left(1,\ldots ,n_{s}\right)\}.$$

In Equation (1), $x_{ri}$ and $x_{sj}$ represent features calculated from patients of EGFR wildtype and mutant, respectively. In addition, *dist*() represents the calculation of the absolute difference between two feature values. $n_{r}$ and $n_{s}$ represent the total number of EGFR wildtype and mutant patients, respectively. In our work, $n_{r}=20$ and $n_{s}=26$.

(2) $$NS_{ij}=\left\{d|d=dist\left(p_{si},p_{sj}\right),s\in \left(1,\ldots ,n_{p}\right)\right\},\;i\in \left(1,\ldots ,n_{c}\right),\;j\in \left(1,\ldots ,n_{c}\right),\;i>j.$$

In Equation (2), $p_{si}$ and $p_{sj}$ represent features calculated from phantom lesions under two different scanning conditions i and j, respectively. $n_{p}$ and $n_{c}$ represent the total number of phantom lesions and scanning conditions, respectively. In our work, $n_{p}=24$, and $n_{c}=8$ (two scanning machines multiply four different effective mAs).

Then, biological and noise signals were normalized by $\mu_{BS}+\sigma_{BS}$ (where $\mu_{BS}$ and $\sigma_{BS}$ represent the mean value and standard deviation computed from the biological signal set BS), and compared by using a two-sample *t*-test. Comparisons were made in an isolated pairwise fashion, e.g., 50 mAs versus 100 mAs. A significant difference in the two distributions, defined using a *p*-value < 0.05, demonstrated that at those two scanning conditions, the biological signal would be unaffected by the noise and maintain its ability to distinguish the two groups. Features that had signal values significantly different from noise values were considered to be robust at those scanning conditions whereas features that had signal values that were not significantly different from noise values were deemed to be not robust at those settings.

All procedures involved in this section were performed in MATLAB 2014a (MathWorks Inc., Natick, MA, USA).

## 3. Results

### 3.1. Feature Selection

The radiomics features selected for being reproducible, non-redundant, and informative are included in Table 2. In total, 741 out of 1160 features had a CCC > 0.85 on the test-retest dataset and were deemed reproducible in the initial screen. Four features, Tumor-Mass, DWT-Energy, Sigmoid-Offset-Mean, and Gabor-Energy had an AUC > 0.7, and were identified to be non-redundant and informative.

Tumor-Mass quantifies the mass of the entire tumor, i.e., given the same volume, a partial-solid tumor has a smaller tumor mass, while a solid tumor has a larger tumor mass. In our study, Tumor-Mass is lower in the EGFR-mutant group, −4.07 ± 6.05 × 10^6^, than in the EGFR-wildtype group, 0.18 ± 3.19 × 10^6^, indicating that tumors in the EGFR-mutant group may consist of a more partial-solid component.

Sigmoid-Offset-Mean quantifies the sharpness of tumor boundary (i.e., intensity transition across the lesion boundary [31]) by using a sigmoid function to fit the tumor boundary in image. Accordingly, a low value indicates a sharp tumor boundary, while a high value indicates a smooth tumor boundary. In our study, Sigmoid-Offset-Mean decreased from 13.12 ± 47.56 in the EGFR-mutant group to −33.34 ± 45.67 in EGFR-wildtype group, suggesting that tumors in EGFR-mutant group have a smoother tumor boundary.

DWT-Energy and Gabor-Energy are two textual features quantifying the intratumoral heterogeneity of the tumor. Large values of DWT-Energy and Gabor-Energy indicate high heterogeneity within the tumor. As shown in Table 2, both DWT-Energy and Gabor-Energy decreased from EGFR-mutant group to EGFR-wildtype group, indicating that EGFR mutant tumors were more heterogenous than EGFR wildtype tumor.

### 3.2. Robustness Validation

**Figure 2:**
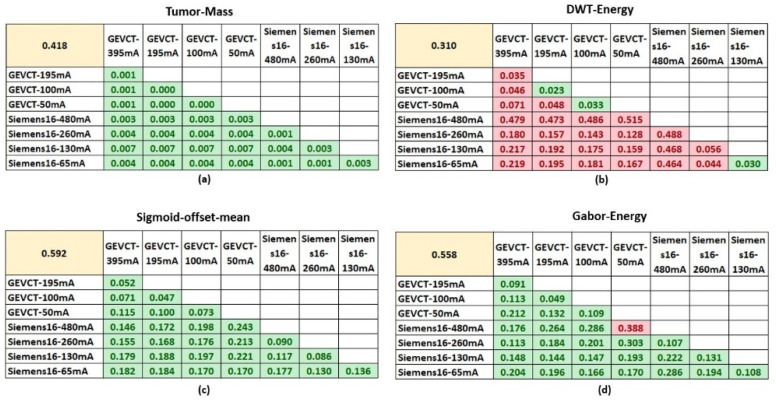
Comparisons of biological and noise signals. The four tables show the comparison of biological and noise signals for each of the 4 non-redundant and informative features. The top left yellow highlighted cell in each table shows the average value of biological signal for that respective feature. The average values of noise signal generated from each specific pairwise setting comparison are shown in the tables. Cells highlighted in green had a *p*-value < 0.05 using the two-sample *t*-test, whereas cells highlighted in red had a *p*-value > 0.05. (**a**–**d**) are the comparisons for the features, Tumor-Mass, DWT-Energy, Sigmoid-offset-mean and Gabor-Energy, respectively.

Three out of the four selected features were demonstrated to be robust and unaffected by the noise generated from using different scanning conditions. They were Tumor-Mass, Sigmoid-Offset-Mean and Gabor-Energy. The comparisons between biological signal and noise signal distributions for the four selected radiomics delta features are detailed in Figure 2.

For Tumor-Mass, the biological signal distribution had a mean value of 0.418 and the noise signal between the pairs of imaging settings had a distribution with mean values no greater than 0.007. In addition, a two-sample *t*-test determined that the noise signal distributions were all significantly different from the biological signal distribution according to a *p*-value of <0.05. Sigmoid-Offset-Mean had a biological signal distribution with a mean value of 0.592 and noise distributions with mean values no greater than 0.25. Similarly, a two-sample *t*-test determined that the biological signal and noise signal distributions were significantly different. This difference suggests that at these settings, the noise variability from using a different scanner or tube current will not significantly impact the true biological signal’s ability to differentiate between the classes of EGFR mutant and EGFR wildtype.

The Gabor-Energy feature had a biological signal distribution with a mean value of 0.558 and noise signal distributions with mean values no greater than 0.388. The majority of noise signal distributions were significantly different from the biological signal distribution. Only a single comparison between Scanner #1-50 mA and the Scanner #2-480 mA had a noise signal distribution that failed to demonstrate a significant difference from the biological signal distribution. In the majority of cases, the Gabor-Energy feature will continue to differentiate between the classes of EGFR mutant and EGFR wildtype.

However, the DWT-Energy feature’s biological signal and random distributions were for the most part, not significantly different. The biological signal distribution had a mean value of 0.310 whereas the noise distribution mean values ranged from 0.02 to 0.49. There were only three instances where the distributions were significantly different, Scanner #1-100 vs. Scanner #1-50 mA, Scanner #1-195 vs. Scanner #1-100 mA and Scanner #2-130 mA vs. Scanner #2-65 mA. Non-significance indicates that the influence of noise has a higher likelihood of impacting the biological signal, and casts doubt on whether that signal retains its ability to distinguish the two classes in those circumstances. Although there were some exceptions, the majority of pairwise setting comparison indicate that this feature was not robust, as it could only generalize to CT images acquired by very similar scanning parameters, e.g., 50 mAs to 100 mAs in Scanner #1 and 65 mAs to 130 mAs in Scanner #2.

## 4. Discussion

Use of radiomics in a clinical setting has long been hampered by repeatability and reproducibility concerns. Feature robustness also needs to be considered in order to maximize model generalizability. The aim of this study was to test a new framework of screening radiomics features for repeatability, reproducibility, and robustness across two scanner types and several different tube currents using phantom lung lesions. This framework can be applied to a wide variety of settings and can point out features that are resistant to the noise generated from different CT imaging acquisition parameters.

Our proposed framework builds upon using a test-retest dataset to screen for reproducibility, a standard feature selection strategy, and adds robustness validation by comparing the magnitude of the biological signal relative to the noise from different CT acquisition parameters, which were obtained using phantom lung lesions. Demonstrating that noise generated from a cross-setting analysis is minimal compared to the biological signal will provide evidence that radiomics features will be robust across those settings. In our pilot example using radiomics to predict EGFR mutation status in patients with NSCLC, we extracted 1160 quantitative features, of which only 741 were considered to be reproducible, and only four were ultimately selected to be non-redundant and informative. In our robustness validation, we found that three out of the four selected features had biological signals that were significantly greater than the noise variation detected across the studied tube currents and scanner types. The DWT-Energy feature failed to show a significant difference between the biological signal distribution and the noise signal distributions. This suggests that noise from the use of different settings may impact the biological signal’s ability to differentiate between the two classes of EGFR mutant and wildtype. As a result, incorporation of this feature in a classification model may limit the model’s performance to only within CTs using the same (or close) tube current and scanner from which it was trained on. On the other hand, demonstration of robustness for the other three features suggest that classification performance will be sustained even in CTs that were obtained using a different tube current or scanner. Theoretically, this means that models built from these robust features will also maintain their performance in cases of external validation where CT acquisition parameters commonly differ from the original training data.

The use of phantom studies to evaluate feature reproducibility has been suggested many times and is a part of the radiomics quality score (RQS) suggested by Lambin et al. [32]. We have previously demonstrated the use of lung and liver phantom lesions to evaluate the effect of parameters such as slice thickness on selected radiomics features [33,34]. This framework adds on to the common use of test-retest datasets for reproducibility and demonstrates a method to determine feature robustness at a wide variety of CT acquisition settings using phantom lung lesions. Use of this framework in future radiomics studies will identify robust features that may contribute to higher generalizability of prediction models, especially in external validation settings in which CT acquisition settings may differ. This may be a critical step towards the future implementation of radiomics in clinical settings.

In our work, univariance analysis was performed instead of multivariance analysis. The reason is that, compared to multivariance analysis, univariance analysis has two advantages: (a) univariance analysis has lower risk of overfitting when performed on a small amount of data; (b) in the field of medical image analysis, univariance analysis is more interpretable and easily understood.

Our study has some limitations. The framework was tested in a case study involving predicting EGFR mutation status in patients with NSCLC and had a relatively small number of patient cases and a single-center design. Our case study also only demonstrated the effect of tube current and two scanner types, but the framework can be applied to any setting or variable in question as well as to other biologic differences and populations. The appropriate use of a variety of phantoms is critical to establishing the true range of noise from different settings, but the use of FDA-customized lung lesion phantoms may not be feasible at all institutions. In addition, we have not demonstrated how selection of robust features will affect model performance in a validation setting with different CT acquisition parameters, although theoretically we expect that it should improve performance. Future work will involve incorporating this framework in radiomics studies with a larger sample size and an external validation dataset to test the potential improvement in performance.

## 5. Conclusions

In conclusion, our study proposes and tests a novel framework for identifying robust radiomics features in a case study predicting tumor treatment response in NSCLC patients after treatment with Gefitinib using phantom lung lesions. We found that even though selected features for classification in this experiment were determined to be reproducible, non-redundant, and informative, not all features were robust to noise effects from the use of different tube currents and scanner types. Our described method may increase the generalizability of prediction models by identifying robust features, but additional studies are needed to assess the true impact of feature robustness on model performance.

## Figures and Tables

**Table 1:** Scan conditions used in CT data acquisition.

Scanning Machine	Scanner #1	Scanner #2
Scanning Parameters		
Tube voltage (kVp)	120	120
Pitch	1.375	1.2–1.3
X-ray Tube Current (mA)	395, 195, 100, 50	480, 260, 130, 65
CTDI_vol_ (mGy)	23.4, 11.5, 5.9, 2.9	14.4, 7.2, 3.6, 1.8
Exposure Time (s)	0.7	0.5
Collimation Configuration	16 × 0.625 = 10 mm	16 × 0.75 = 12 mm
Reconstruction kernel	LUNG	B70f
Slice Interval (mm)	1.25	0.9
Slice Thickness (mm)	1.25	1
Pixel Spacing (mm)	0.7	0.7

Scanner #1 refers to the GE Lightspeed Pro model and Scanner #2 refers to the Siemens Sensation 16 model.

**Table 2:** Identified non-redundant and informative radiomics features.

Feature Name	AUC (95% CI)	Feature Distribution on the EGFR Mutant Group (Mean ± Std)	Feature Distributionon the EGFR Wildtype Group (Mean ± Std)
**Tumor-Mass**	0.865 (0.605, 0.937)	−4.07 ± 6.05 × 10^6^	0.18 ± 3.19 × 10^6^
**DWT-Energy**	0.821 (0.562, 0.934)	1.02 ± 1.73 × 10^9^	−2.45 ± 9.20 × 10^9^
**Sigmoid-Offet-Mean**	0.760 (0.529, 0.918)	13.12 ± 47.56	−33.34 ± 45.67
**Gabor-Energy**	0.738 (0.520, 0.887)	1.06 ± 2.01 × 10^5^	−0.42 ± 1.98 × 10^5^
